# MRI‐guided attenuation correction in torso PET/MRI: Assessment of segmentation‐, atlas‐, and deep learning‐based approaches in the presence of outliers

**DOI:** 10.1002/mrm.29003

**Published:** 2021-09-04

**Authors:** Hossein Arabi, Habib Zaidi

**Affiliations:** ^1^ Division of Nuclear Medicine and Molecular Imaging Geneva University Hospital Geneva Switzerland; ^2^ Geneva University Neurocenter Geneva University Geneva Switzerland; ^3^ Department of Nuclear Medicine and Molecular Imaging University of Groningen University Medical Center Groningen Groningen Netherlands; ^4^ Department of Nuclear Medicine University of Southern Denmark Odense Denmark

**Keywords:** body truncation, deep learning, metal artifact, PET/MRI, quantitative PET

## Abstract

**Purpose:**

We compare the performance of three commonly used MRI‐guided attenuation correction approaches in torso PET/MRI, namely segmentation‐, atlas‐, and deep learning‐based algorithms.

**Methods:**

Twenty‐five co‐registered torso ^18^F‐FDG PET/CT and PET/MR images were enrolled. PET attenuation maps were generated from in‐phase Dixon MRI using a three‐tissue class segmentation‐based approach (soft‐tissue, lung, and background air), voxel‐wise weighting atlas‐based approach, and a residual convolutional neural network. The bias in standardized uptake value (SUV) was calculated for each approach considering CT‐based attenuation corrected PET images as reference. In addition to the overall performance assessment of these approaches, the primary focus of this work was on recognizing the origins of potential outliers, notably body truncation, metal‐artifacts, abnormal anatomy, and small malignant lesions in the lungs.

**Results:**

The deep learning approach outperformed both atlas‐ and segmentation‐based methods resulting in less than 4% SUV bias across 25 patients compared to the segmentation‐based method with up to 20% SUV bias in bony structures and the atlas‐based method with 9% bias in the lung. The deep learning‐based method exhibited superior performance. Yet, in case of sever truncation and metallic‐artifacts in the input MRI, this approach was outperformed by the atlas‐based method, exhibiting suboptimal performance in the affected regions. Conversely, for abnormal anatomies, such as a patient presenting with one lung or small malignant lesion in the lung, the deep learning algorithm exhibited promising performance compared to other methods.

**Conclusion:**

The deep learning‐based method provides promising outcome for synthetic CT generation from MRI. However, metal‐artifact and body truncation should be specifically addressed.

## 1 INTRODUCTION

Hybrid imaging in the form of PET/CT or PET/MRI is one of the modern and promising tools witnessed to empower a deeper understanding of the hallmarks of cancer.^1^ Since the emergence of hybrid PET/MR imaging, a countless number of approaches have been introduced to tackle the challenges of MRI‐guided attenuation correction (AC) to achieve the full quantitative potential of PET imaging.^2^ Earlier approaches in this regard relied on bulk tissue segmentation,^3^, ^4^ maximum likelihood reconstruction of attenuation and activity (MLAA),^5^, ^6^ and atlas‐based synthetic CT (sCT) generation.^7^, ^8^, ^9^, ^10^ More recently, with the revolution induced by the introduction of deep learning approaches,^11^ MRI‐based sCT generation using one of the state‐of‐the‐art architectures of convolutional neural networks became the dominant trend or mainstream in this field.^12^, ^13^, ^14^, ^15^, ^16^, ^17^ Moreover, the exceptional capability of deep learning algorithms in providing satisfactory solutions to inverse problems has spurred novel frameworks for PET AC that were not feasible (or at least not providing a comparable performance) with other approaches. These frameworks include AC in the image domain,^18^, ^19^, ^20^ AC map estimation from uncorrected PET (PET‐nonAC) images,^21^ estimation of AC factors from time‐of‐flight (TOF) PET data,^22^ and combination of MLAA reconstruction with deep learning‐based approaches.^23^

Synthetic CT generation from MRI is of special interest, not only for AC in PET/MRI but also in MRI‐guided radiation planning.^12^, ^24^ Online adaptation of radiation treatment plans based on MRI requires reliable estimation of electron density from MRI.^25^ A large body of literature reported on state‐of‐the‐art deep learning‐based algorithms for sCT generation from MRI.^13^, ^16^ Qualitative and quantitative assessments have demonstrated the reliable performance of deep learning‐based algorithms for sCT generation, wherein clinically acceptable average errors of 5% in PET AC^15^, ^26^ and 2% in RT planning^27^ were achieved. The bulk of research in MRI‐guided sCT generation focused on the development of novel algorithms to enhance the performance of existing approaches^14^ or the comparison of different methods using large cohort of patients.^15^ These studies commonly report the overall performance of these evaluated techniques through the mean error measured over a clinical or simulated dataset. As such, investigation of outliers or potential sources of algorithms failure has not been sufficiently addressed.

In this work, instead of developing “yet another” deep learning‐based sCT generation algorithm or conducting a comparative assessment of the performance of various approaches, we set out to examine cases where the input subjects bear some sort of abnormalities and explore if they induce outliers or failures. Hence, the focus is on the torso sCT generation from MRI, wherein a deep learning‐based algorithm is evaluated for abnormal cases, which may not be present in the training dataset. For comparison, other commonly used sCT generation methods, including atlas‐ and segmentation‐based approaches were also implemented to provide a baseline to assess the performance of the deep learning‐based method. These sCT generation approaches were examined for abnormal input subjects, which might occur in routine clinical practice. Four different specific cases, including MR body truncation, MR susceptibility to metallic artifacts, odd anatomy, and small malignant lesions in the lung were studied. The performance of the deep learning method and the other two sCT generation approaches was specifically investigated for these cases wherein an MR image bearing one of the aforementioned abnormalities was considered as input to the sCT generation methods.

## 2 METHODS

### 2.1 Data acquisition

The patient cohort used in this study consisted of 25 torso ^18^F‐FDG PET/MRI and PET/CT scans performed for staging or as follow‐up examination of head and neck malignancies. Patients underwent PET/MRI examinations followed by PET/CT scanning using a single radiotracer injection. The institution’s ethics committee approved the study protocol. PET/MR imaging was performed on the Ingenuity TF PET/MRI system (Philips Healthcare, Cleveland, OH),^28^ wherein torso MR Dixon (3D volumetric interpolated T1‐weighted sequence) images were acquired as part of the routine clinical protocol. The MR Dixon acquisition was performed using a flip angle of 10°, TE of 11.1 ms, field of view (FOV) of 450 × 354 mm^2^, and 0.85 × 0.85 × 3 mm^3^ voxel size. MR Dixon imaging results in water‐only, fat‐only, out‐phase, and in‐phase (IP) MR images. The IP Dixon images were used for evaluation of the MR‐guided approaches for sCT generation.

^18^F‐FDG PET/CT scans were performed shorty after PET/MRI examination on a Biograph 64 True Point scanner (Siemens Healthcare, Erlangen, Germany). PET acquisitions (for the PET/CT scan) were performed in five or six bed positions (3 min per bed) (covering the skull base to the lower abdomen) 126 ± 10 min post‐injection of 369 ± 22 MBq^18^F‐FDG. Unenhanced CT images acquired at 120 kVp, 180 mAs with a pitch of 1.2 were used for PET AC. The PET raw data from the PET/CT scans were used to reconstruct PET images using the different synthetic CT images as AC maps. This was performed using 3D ordinary Poisson‐ordered subset expectation maximization (OP‐OSEM) algorithm with four iterations and eight subsets implemented within the offline e7 tool (Siemens Healthcare, Knoxville, TN).

Due to the time gap between MR Dixon and CT image acquisition, the images were co‐registered to put the MR images into PET/CT spatial coordinates (both PET/CT and PET/MR images were acquired with arms down). Image alignment was carried out using a combination of affine and non‐rigid image registration using the normalized mutual information as loss function and adaptive stochastic gradient descent for the optimization process with B‐spline interpolator. For cases with significant misalignment errors, the registration procedure was repeated a couple of times with manual adjustment of the elasticity parameters to achieve the desired results.^29^ Dixon IP images were deformably registered to the corresponding CT images using the Elastix package (based on the ITK library), where patients presenting with large misalignments through visual inspection were semi‐manually adjusted to achieve satisfactory results.

Prior to image alignment, all MR images underwent N4 bias correction and histogram matching to reduce intra‐subject non‐uniformity of the low‐frequency intensities and inter‐subject intensity variation, respectively. As a result of image registration, all MR images were transformed to the reference CT spatial coordinates at a voxel size of 1.36 × 1.36 × 2.5 mm^3^. After the image registration process, all CT and MR images were normalized to an intensity range of 0 to 1. The training, implementation, and evaluation of the synthetic CT generation approaches were performed using the same matrix and voxel sizes for both MR and CT images. MR images with severe body truncation, metal‐induced artifacts as well as patients with odd anatomy or small malignant lung nodules were selected for the assessment of potential outliers in connexion with sCT generation methods (Supporting Information Table S1, which is available online).

### 2.2 Deep learning‐based approach

A residual deep convolutional neural network was used to learn end‐to‐end MRI to CT conversion to generate torso sCT from Dixon MRI sequences. The residual deep learning model, referred to as HighResNet,^30^ implemented in Python library of the Niftynet platform,^31^ was configured in a 2D manner to convert transaxial slices of the IP Dixon images into corresponding sCT slices. A number of deep learning models were implemented/compared for synthetic CT estimation from MR images, wherein the HighResNet model exhibited superior performance, hence motivating its selection for this study (Supporting Information Table S2).^32^, ^33^ HighResNet consists of 20 convolutional layers wherein the first seven layers are equipped with 3 × 3 × 3 voxel convolution kernels. The first seven layers were designed to depict the low‐level image features from the input images. The next seven layers are responsible for extraction of the medium‐level features via dilation of the convolution kernel by a factor of two. The last six layers, which use a dilation factor of four, capture the high‐level image features. Every two convolutional layers are connected via a residual connection. In the residual blocks, the convolutional layers are coupled with batch normalization and element‐wise rectified linear unit (ReLU). The HighResNet network benefits from several residual blocks to conduct an effective training without adding to the complexity of the network or increasing the number of trainable parameters. To this end, residual or shortcut connections are established to deactivate/skip certain layers in the architecture to create a fluid/consistent propagation of the information/features across the different layers to avoid gradient vanishing. The residual connections are intended to transmit signals (and/or the extracted features from the input data or measured errors from the reference/target) forward and backward within the layers/blocks. The HighResNet model, in comparison with other similar architectures, has the merit of high resolution (using dilated convolution kernels instead of max‐pooling layers) image processing and feature extraction within the different layers of the network, which render this architecture a powerful model for image translation/regression and segmentation/classification tasks.^30^ Although the HighResNet model was previously used for synthetic CT generation in head and pelvis imaging or PET AC,^32^, ^34^, ^35^, ^36^ this work used this model for the first time for synthetic CT generation from torso MR images.

The training of the network was carried out using a five‐fold cross‐validation scheme. At each validation step, 20 subjects were used for the training of the model whereas 5 unseen subjects were used for model validation/testing. As a result, five different deep learning models were built and evaluated on the unseen validation dataset. The results were reported for the validation dataset unseen by the models. The L2‐norm loss function along with Adam optimization were used to train the model. The learning rate varied from 0.04 to 0.01 according to the recommendations in Ref. [37]. Prior to training, MR images were resampled to the resolution of CT images (0.98 × 0.98 × 1.5 mm^3^), and a batch size of 32 slices was used within the training of the model.

### 2.3 Atlas‐based approach

A state‐of‐the‐art atlas‐based method relying on a local weighting scheme was implemented for comparison with the deep learning‐based sCT generation approach.^38^, ^39^ To this end, the IP images and the corresponding co‐registered CT images were arranged in atlas and target datasets following a leave‐one‐out‐cross‐validation (LOOCV) scheme. The IP images in the atlas dataset were deformed to the target IP image via a combination of affine and non‐rigid image registration using normalized mutual information as cost function and B‐spline function as interpolator. The open source Elastix software was used for image registration following the procedure described in Ref. [40]. Given the transformation matrices obtained from atlas to target MR registration, the corresponding CT images were mapped to the target IP images.

To generate atlas‐based sCT images, in the first step, bone segmentation was optimized on the target IP image via a voxel‐wise atlas voting scheme. The output of this step is a binary bone map, which will be considered as the most probable bone map of the target IP image.

In the next step, the obtained bone map for the target IP image is used to generate the weighting factors for each subject in the atlas dataset through a voxel‐wise comparison of the signed distance maps. The final continuous valued sCT image was generated through a voxel‐wise weighting scheme based on the phase congruency map (PCM)^43^ morphology likelihood between the IP images in the atlas dataset and the target IP image. Further details about the atlas‐based method is provided in Ref. [38].

### 2.4 Segmentation‐based approach

The segmentation‐based method implemented on the Ingenuity TF PET/MRI system (Philips Healthcare, Cleveland, OH) was used in this work for comparison with the two above referenced methods.^28^, ^41^ This segmentation‐based approach (bulk tissue classification) carries out a body contour delineation on the MR image to discriminate background air from the body volume. Then, the lung tissue is segmented from the soft‐tissue (rest of the body). This segmentation approach leads to a three‐class attenuation map where attenuation coefficients of 0.096 cm^−1^ (0 HU), 0 cm^−1^ (−1000 HU), and 0.022 cm^−1^ (−770 HU) are assigned to soft‐tissue, background air, and lung, respectively.

### 2.5 Evaluation strategy

For quantitative assessment of the MRI‐guided sCT generation approaches, PET data of the 25 patients were reconstructed four times using: (i) CT‐based AC map serving as standard of reference (PET/CT), (ii) the three‐class AC map obtained from the segmentation‐based method (PET‐Seg), (iii) atlas‐based sCT (PET‐Atlas), and (iv) deep learning‐based sCT AC map (PET‐DL). Considering PET/CT images as reference,^42^ quantitative analysis of radiotracer uptake in PET‐Seg, PET‐Atlas and PET‐DL was performed for the different tissues, namely lung, bone, and soft‐tissue using Equation (1).

(1) $$Bias\;\left(\%\right)=\frac{PET\_sCT\left(\mathrm{SUV}\right)‐PET\_CT\left(\mathrm{SUV}\right)}{PET\_CT\left(\mathrm{SUV}\right)}\times 100\%.$$

The intensity values in all PET images were converted to standardized uptake value (SUV) for quantitative assessment. Bones and soft‐tissues within the body were segmented from reference CT images using the following intensity‐based thresholds for soft‐tissue (between −400 HU and 160 HU) and bone (> 160 HU) while the lung regions were manually defined on CT images. To avoid artificially increased bias, voxels with extremely low SUVs were ignored in the voxel‐wise calculation of quantification bias using an empirical threshold of 0.05 SUV. Organ‐wise assessment of SUV bias was performed for the different approaches within the right and left lungs, cerebellum, aorta cross, liver, spleen, and cervical bone 5&6. Moreover, 35 volumes of interest (VOIs) were manually drawn on the malignant lesions for quantitative analysis of the different MRI‐guided PET AC techniques. The VOIs were drawn manually by a nuclear medicine physician on regions of abnormally increased tracer uptake identified as malignant lesions. Supporting Information Figure S1 depicts representative VOIs drawn on abnormally increased radiotracer uptake in the head and neck area. No more than 2 VOIs were defined on a single patient. The majority of the malignant lesions resided in the head and neck area (23 lesions), 7 lesions in the liver, and 5 lesions in the lung. The size of lesions (or the VOIs drawn on lesions) ranged from 0.5 to 1.9 mL. Moreover, the root mean square error (RMSE) was calculated between PET‐DL, PET‐Atlas, and PET‐Seg and reference PET‐CTAC images for each subject using Equation (2) where *v* and *i* are the total number of voxel and voxel index, respectively.

(2) $$RMSE=\sqrt{\frac{\sum_{i=1}^{V}{\left(PET\_CT\left(i\right)‐PET\_sCT\left(i\right)\right)}^{2}}{V}}.$$

The differences between the quantitative metrics calculated over the PET‐Seg, PET‐Atlas, and PET‐DL images were investigated using the paired t‐test method where *P*‐values smaller than 0.05 were considered as statistical significance.

Apart from overall performance assessment of the segmentation‐, atlas‐, and deep learning‐based approaches, the main aim of this study was to investigate how these methods behave when the input images bear some sort of abnormalities and as such, are different from the normal population. To this end, MR images with severe body truncation, metal‐artifact, abnormal anatomy, and small malignant lesions in the lung were carefully assessed. Each of these cases will be separately addressed in the Results and Discussion sections.

## 3 RESULTS

Table 1 summarizes the overall quantitative performance of the segmentation‐, atlas‐, and deep learning‐based methods for the three main tissues within the body. The deep learning method resulted in less 5% SUV bias, demonstrating its superior accuracy compared to the other methods. The *P*‐values calculated between the results associated with the PET‐Seg and the other two images (PET‐Atlas and PET‐DL) were all statistically significant with *p*‐values < 0.01. The *p*‐values reported in Tables 1 and 2 were calculated between PET‐Atlas and PET‐DL images. Likewise, the SUV bias estimated over the 35 VOIs drawn on the malignant lesions confirmed the overall better performance of the deep learning method, wherein a mean absolute error of less than 4% was achieved by the PET‐DL approach compared to 5.6% and 10.1% by PET‐Atlas and PET‐Seg techniques reported in Table 3, respectively. Quantitative accuracy of the estimated CT values for major tissue classes by the different synthetic CT generation methods are presented in Supporting Information Table S3.

**TABLE 1:** Relative mean ± SD (absolute mean ± SD) of SUV bias calculated in the lung, soft‐tissue, and bone in PET images corrected for attenuation using the different sCT images

	Lung	Soft‐tissue	Bone
	Mean ± SD	Mean ± SD	Mean ± SD
	(absolute mean ± SD)	(absolute mean ± SD)	(absolute mean ± SD)
PET‐Seg	13.8 ± 8.3	−6.7 ± 5.2	−20.2 ± 11.3
	(15.6 ± 6.8)	(11.9 ± 6.5)	(21.4 ± 9.8)
PET‐Atlas	7.2 ± 5.6	−4.0 ± 4.9	−7.5 ± 4.9
	(9.6 ± 4.4)	(6.3 ± 3.6)	(8.1 ± 3.2)
PET‐DL	−3.7 ± 5.5	2.0 ± 3.9	1.1 ± 3.1
	(4.1 ± 4.2)	(3.1 ± 3.0)	(3.2 ± 2.9)
*P*‐value	0.01	0.03	0.01

Note

PET/CT images were considered as reference. The *P*‐values were calculated between the results associated with PET‐Atlas and PET‐DL images.

**TABLE 2:** Relative mean ± SD (absolute mean ± SD) of SUV bias calculated within the different organs in PET images corrected for attenuation using the different sCT images

Region	PET‐Seg	PET‐Atlas	PET‐DL	*P*‐Value
Right lung	13.3 ± 10.3	6.9 ± 5.8	−2.9 ± 3.6	<0.01
Left lung	15.1 ± 8.4	7.8 ± 7.1	−4.1 ± 3.8	<0.01
Cerebellum	−13.3 ± 6.5	−5.9 ± 5.3	2.1 ± 3.7	<0.02
Aorta cross	−15.9 ± 7.9	−5.5 ± 6.7	1.8 ± 4.0	<0.05
Liver	−9.9 ± 6.4	−8.1 ± 4.6	1.9 ± 3.9	<0.02
Spleen	−10.2 ± 9.7	−5.9 ± 8.1	2.0 ± 4.0	<0.05
Bone (cervical 5&6)	−20.0 ± 9.1	8.3 ± 9.7	3.3 ± 4.3	<0.05

Note

PET/CT images were considered as reference. The *P*‐values were calculated between the results associated with PET‐Atlas and PET‐DL images.

**TABLE 3:** Relative mean ± SD (absolute mean ± SD) of SUV bias calculated in malignant lesions (35 VOIs) in PET images corrected for attenuation using the different sCT images

	PET‐Seg	PET‐Atlas	PET‐DL
	Mean ± SD	Mean ± SD	Mean ± SD
	(absolute mean ± SD)	(absolute mean ± SD)	(absolute mean ± SD)
Lesions	−9.4 ± 6.1	−3.1 ± 4.3	1.1 ± 2.9
	(10.1 ± 5.2)	(5.6 ± 3.5)	(3.4 ± 2.2)
*P*‐value	PET‐Seg vs PET‐Atlas	PET‐Atlas vs PET‐DL	PET‐DL vs PET‐Seg
	0.01	0.03	<0.01

Note

PET/CT images were considered as reference.

The atlas‐based method required 8 h to generate a single synthetic CT image on an Intel® Core ™ i9 11900F system (considering 24 atlas alignments). Conversely, the deep learning method required 15 sec to infer a synthetic CT image after training, with the training of each model (each validation fold) taking approximately 18 h on an NVIDIA GEFORCE RTX 2080 Ti platform.

**FIGURE 1:**
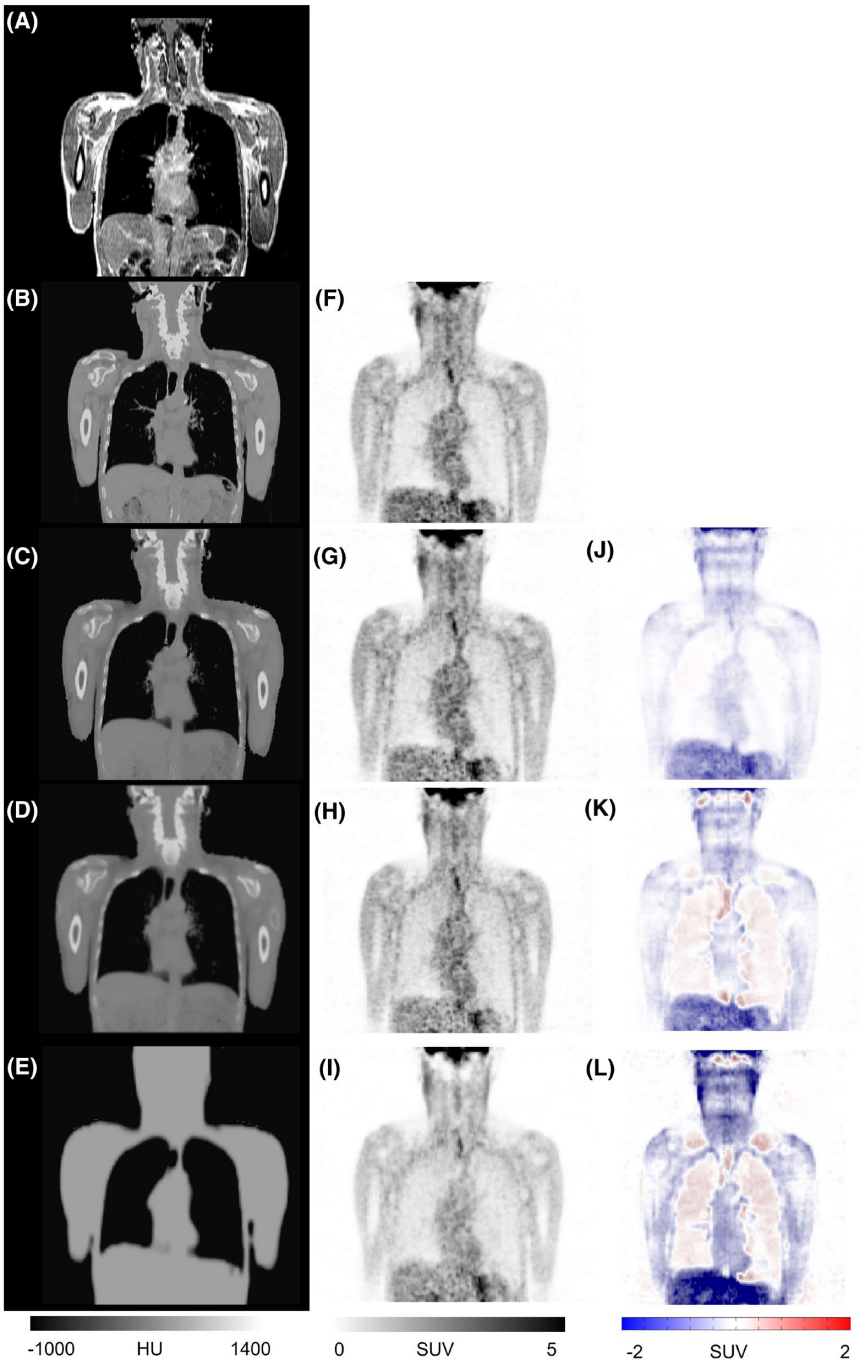
Representative clinical study showing sCT images generated by the different methods and the corresponding PET images. Target MRI (A), reference CT image (B), sCT images generated by the deep learning (C), atlas‐based (D), and segmentation‐based (E) methods. PET‐CT (F), PET‐DL (G), PET‐Atlas (H), and PET‐Seg (I), and their corresponding difference maps PET‐DL – PET‐CT (J), PET‐Atlas – PET‐CT (K), and PET‐Seg – PET‐CT (L)

**FIGURE 2:**
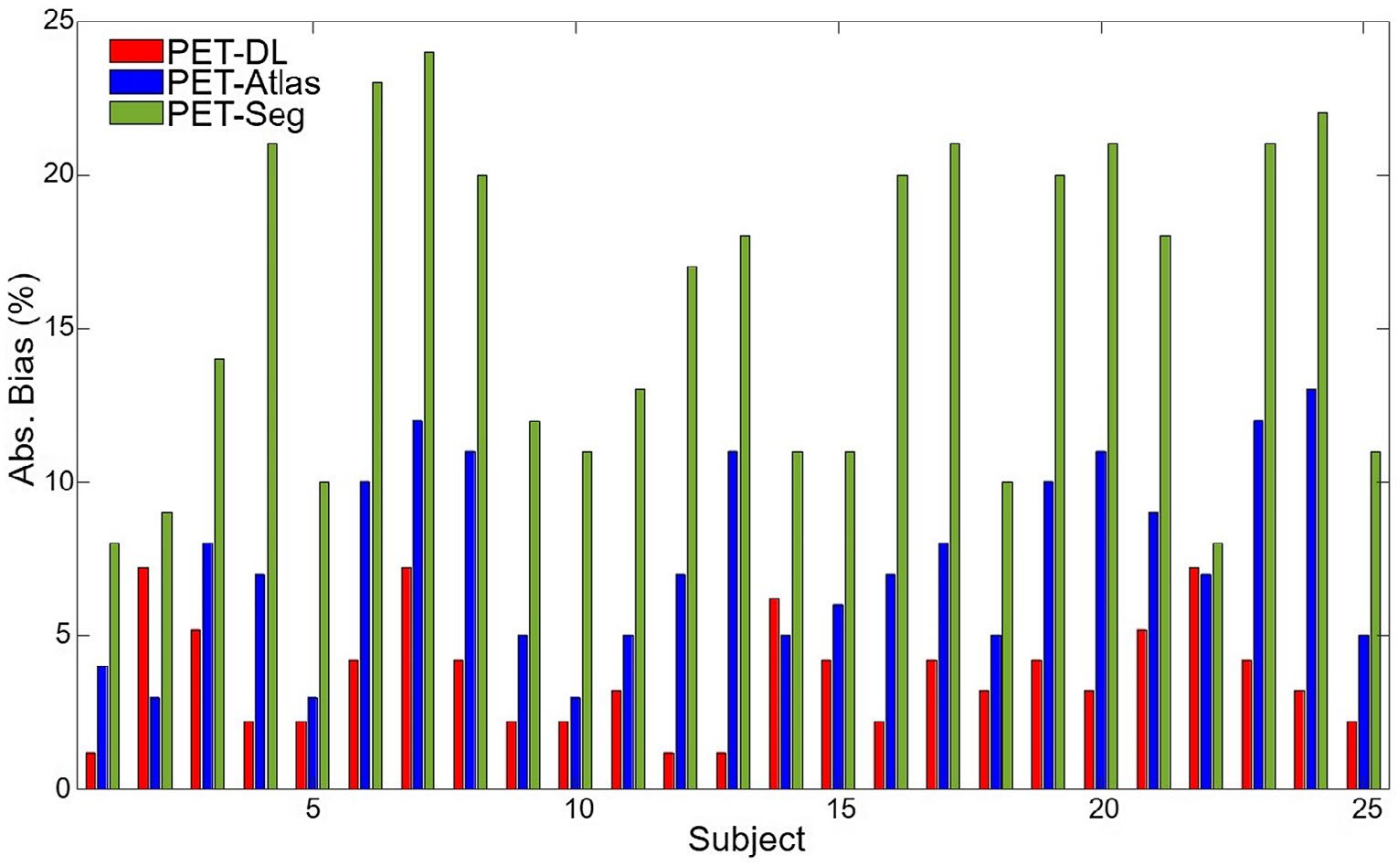
Absolute bias (Abs. Bias (%)) between PET‐DL, PET‐Atlas, and PET‐Seg and reference PET‐CTAC images for each subject

**FIGURE 3:**
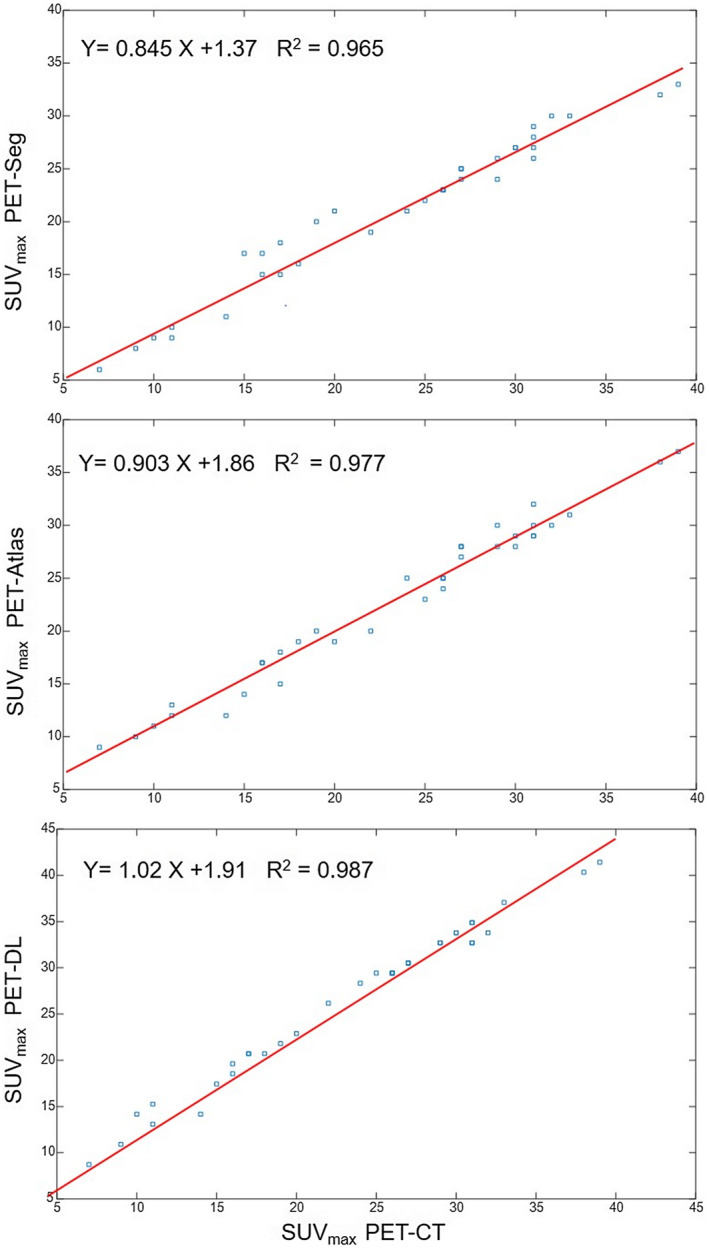
Scatter plots of SUV_max_ calculated in the 35 VOIs drawn on malignant lesions for the different AC techniques

Figure 1 depicts representative coronal views of MR, reference CT and sCT images generated by the segmentation‐, atlas‐, and deep learning‐based approaches. The corresponding PET images along with the difference bias maps (*PET‐sCT* – *PET‐CT*) are also presented. The visual inspection revealed sharper anatomical details extracted by the deep learning‐based method in comparison with the atlas‐based approach. Although the deep learning algorithm exhibited overall superior performance to the other methods, this study focused on how these approaches behave when the input subjects bear some abnormalities, thus deviating from the normal population. The absolute bias (%) calculated between PET‐DL, PET‐Atlas, and PET‐Seg and reference PET‐CTAC images are depicted in Figure 2 for each subject. Moreover, the scatter plots of SUV_max_ calculated within the 35 VOIs drawn on the malignant lesions of PET‐Seg, PET‐Atlas, and PET‐DL images are illustrated in Figure 3.

**FIGURE 4:**
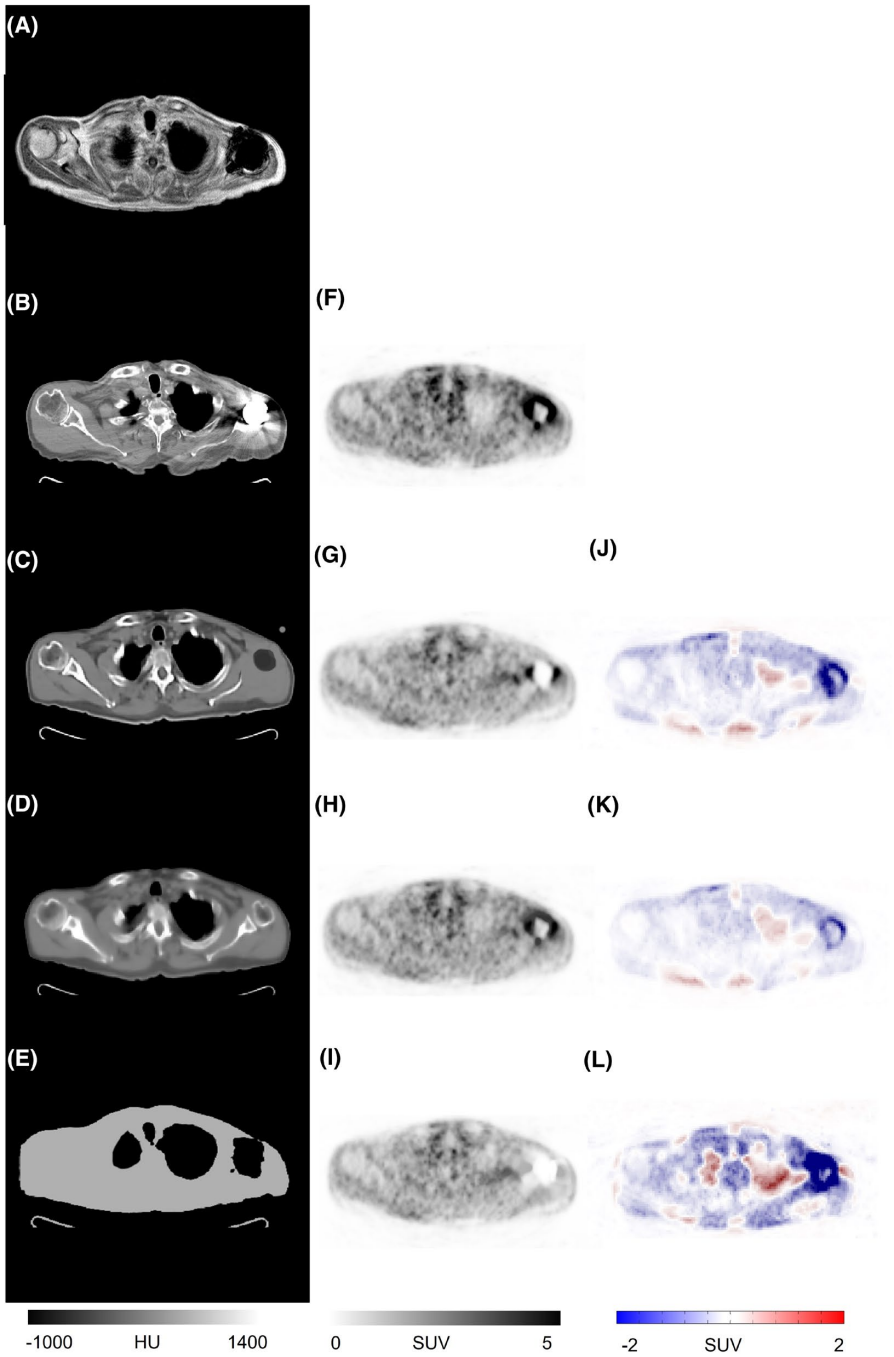
A clinical study presenting with severe metal artifacts. Target MRI (A), reference CT image (B), sCT images generated by the deep learning (C), atlas‐based (D), and segmentation‐based (E) approaches. PET‐CT (F), PET‐DL (G), PET‐Atlas (H), and PET‐Seg (I), and their corresponding difference maps PET‐DL – PET‐CT (J), PET‐Atlas – PET‐CT (K), and PET‐Seg – PET‐CT (L)

For the first case, a subject with severe metal artifacts (shoulder joint prostheses) was studied. Figure 4 depicts an example of an MR image (IP) severely affected by the presence of a metallic object leading to a large void area. This metal‐induced void in the MR image led to mis‐segmentation of the tissue classes in the segmentation‐based sCT (Figure 4E) and consequently large quantitative bias in the corresponding PET‐Seg image (Figure 4I). The void area in the input MR image also gave rise to sub‐optimal performance of the deep learning approach resulting in an incomplete sCT and consequently noticeable SUV bias in the corresponding PET‐DL images (Figure 4G,J). In this regard, the atlas‐based method outperformed the other methods, achieving visually more realistic sCT images (not affected by the metal‐artifact in the MR image) and better quantitative accuracy. The mean SUV bias in the affected area (the entire area with severe metallic artifacts) calculated on PET‐DL, PET‐Atlas, and PET‐Seg images was −38.9%, −11.1%, and −55.6%, respectively. Due to the presence of metallic artifacts in CT images, the reference PET‐CTAC image (prior to metal artifact reduction [MAR]) is also affected.^43^ Considering PET images corrected for attenuation using CT images corrected for metal artifacts using the normalized MAR (NMAR) technique^44^ as reference, the SUV bias for the different approaches turned out to be PET‐DL: −28.3%, PET‐ Atlas: −6.2%, and PET‐Seg: −41.4%. In addition to the metal artifact case depicted in Figure 4 (most severe case), four other cases with significant metal‐induced artifacts were observed in the dataset. Since these cases were caused by dental implants, less MR volumes were affected due to the presence of metal implants and consequently, less quantitative bias was observed in the corresponding PET images. Nevertheless, the different synthetic CT generation approaches exhibited similar performance (Figure 4).

**FIGURE 5:**
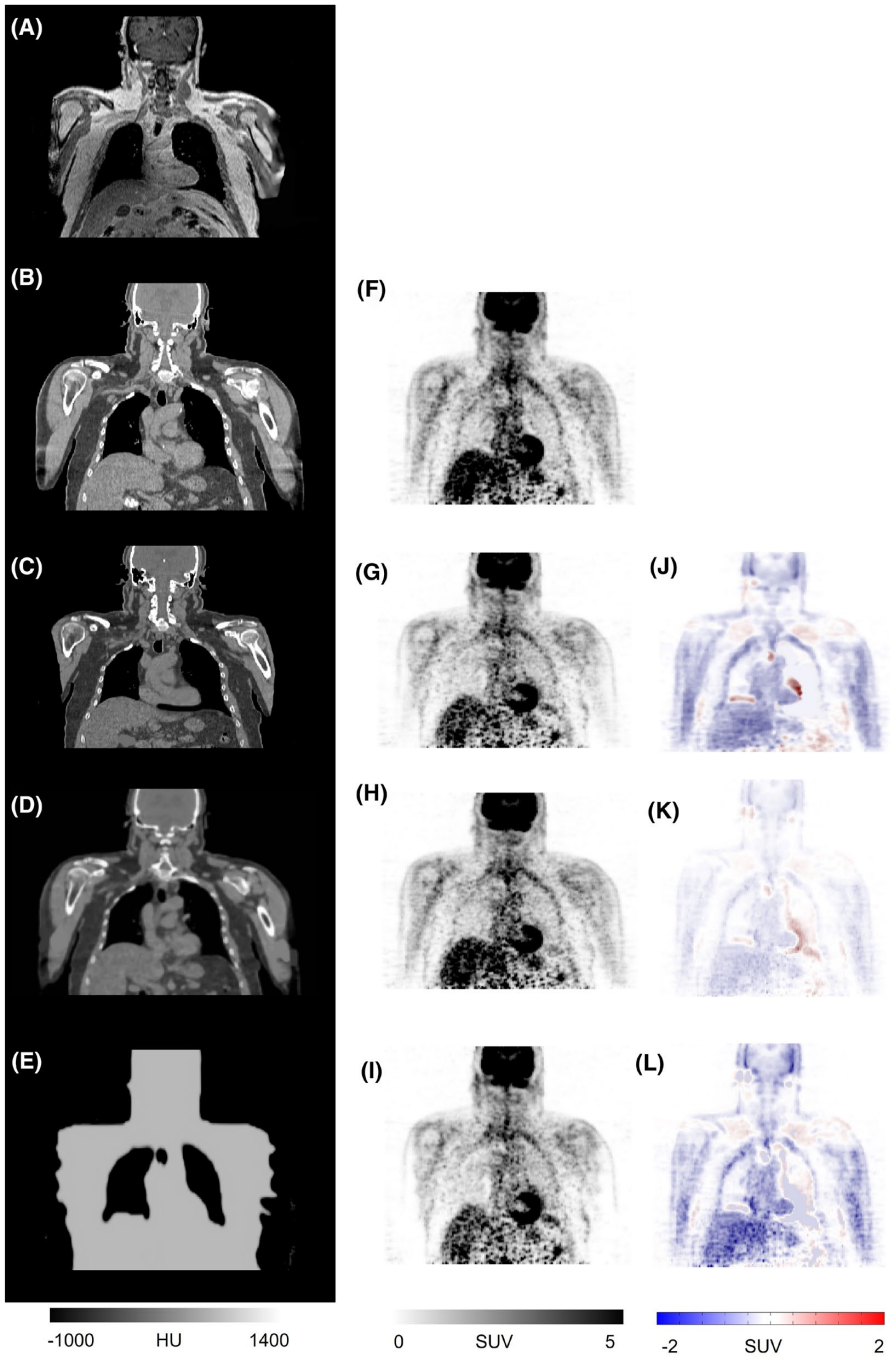
A clinical study presenting with severe body truncation. A, Target MRI. B, reference CT image. sCT images generated by the deep learning (C), atlas‐based (D), and segmentation‐based (E) methods. PET‐CT (F), PET‐DL (G), PET‐Atlas (H), and PET‐Seg (I), and their corresponding difference maps PET‐DL – PET‐CT (J), PET‐Atlas – PET‐CT (K), and PET‐Seg – PET‐CT (L)

Figure 5 depicts an example of severe body truncation, wherein a noticeable part of the arms are missing in the original MR image. This truncation is directly reflected on the segmentation‐based sCT AC map (Figure 5E), hence resulting in a large SUV bias in the PET‐Seg image, particularly in the arm regions (Figure 5I,L). The MRI truncation also affected the deep learning‐based method leading to incomplete AC map (Figure 4C) and considerable bias in the corresponding PET image (Figure 5G,J). In this regard, the atlas‐based approach exhibited superior performance since the resulting AC map (Figure 5D) does not reflect body truncation in the input MR image and thus, no noticeable SUV bias was observed in the corresponding PET‐Atlas image (Figure 5H,K). The mean SUV bias calculated within the truncated area (Figure 5) in PET‐DL, PET‐Atlas, and PET‐Seg images was −41.7%, −8.3%, and −50.0%, respectively. The dataset involved three other cases presenting with body truncation, but less severe cases with less affected/missing volume (Figure 5 depicts the most serious case). The different CT synthetic generation approaches exhibited similar performance to the other cases (Figure 5). It should be noted that due to the less missing/affected volumes in MR images, PET images were less quantitatively affected owing to imperfect AC maps.

**FIGURE 6:**
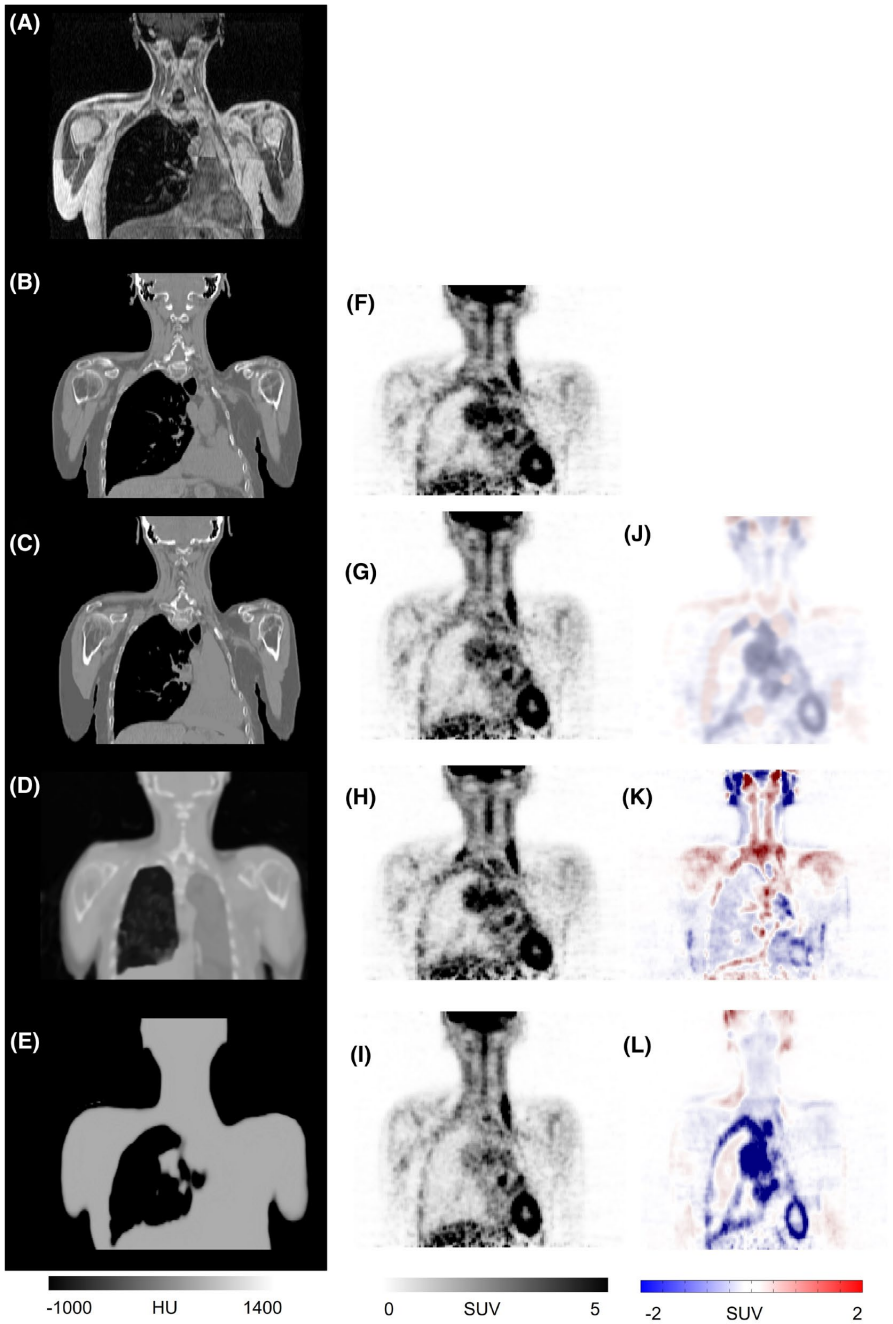
A clinical study presenting with removed left lung. Target MRI (A), reference CT image (B), sCT images generated by the deep learning (C), atlas‐based (D), and segmentation‐based (E) methods. PET‐CT (F), PET‐DL (G), PET‐Atlas (H), and PET‐Seg (I), and their corresponding difference maps PET‐DL – PET‐CT (J), PET‐Atlas – PET‐CT (K), and PET‐Seg – PET‐CT (L)

Severe metal artifact and body truncation are commonly observed in routine MR imaging, whereas patients presenting with abnormal anatomical variability are less likely to occur. In Figure 6, a patient with removed left lung is presented along with the sCTs generated using the different methods. The segmentation‐based method detected appropriately the lung area and resulted in a reasonable AC map (Figure 6E). The atlas‐based method was not able to adapt to the abnormal anatomy in the target MR images, thus resulting in erroneous sCT images (Figure 6D). In this regard, the deep learning‐based method exhibited promising performance through generating a sCT image appropriately reflecting the abnormal anatomy in the target MR image, and consequently minimal SUV bias. The mean SUV bias calculated within the left lung including the heart (Figure 6) in PET‐DL, PET‐Atlas, and PET‐Seg images was −5.4%, −29.7%, and −35.1%, respectively. The segmentation‐based approach assigns a predefined attenuation coefficient to the whole lung tissue across all patients, which led to overall overestimation of PET tracer uptake. However, for the particular patient illustrated in Figure 6, the lung tissue was underestimated by the fixed attenuation coefficient, which resulted in considerable negative PET quantification bias in the vicinity of the lung.

**FIGURE 7:**
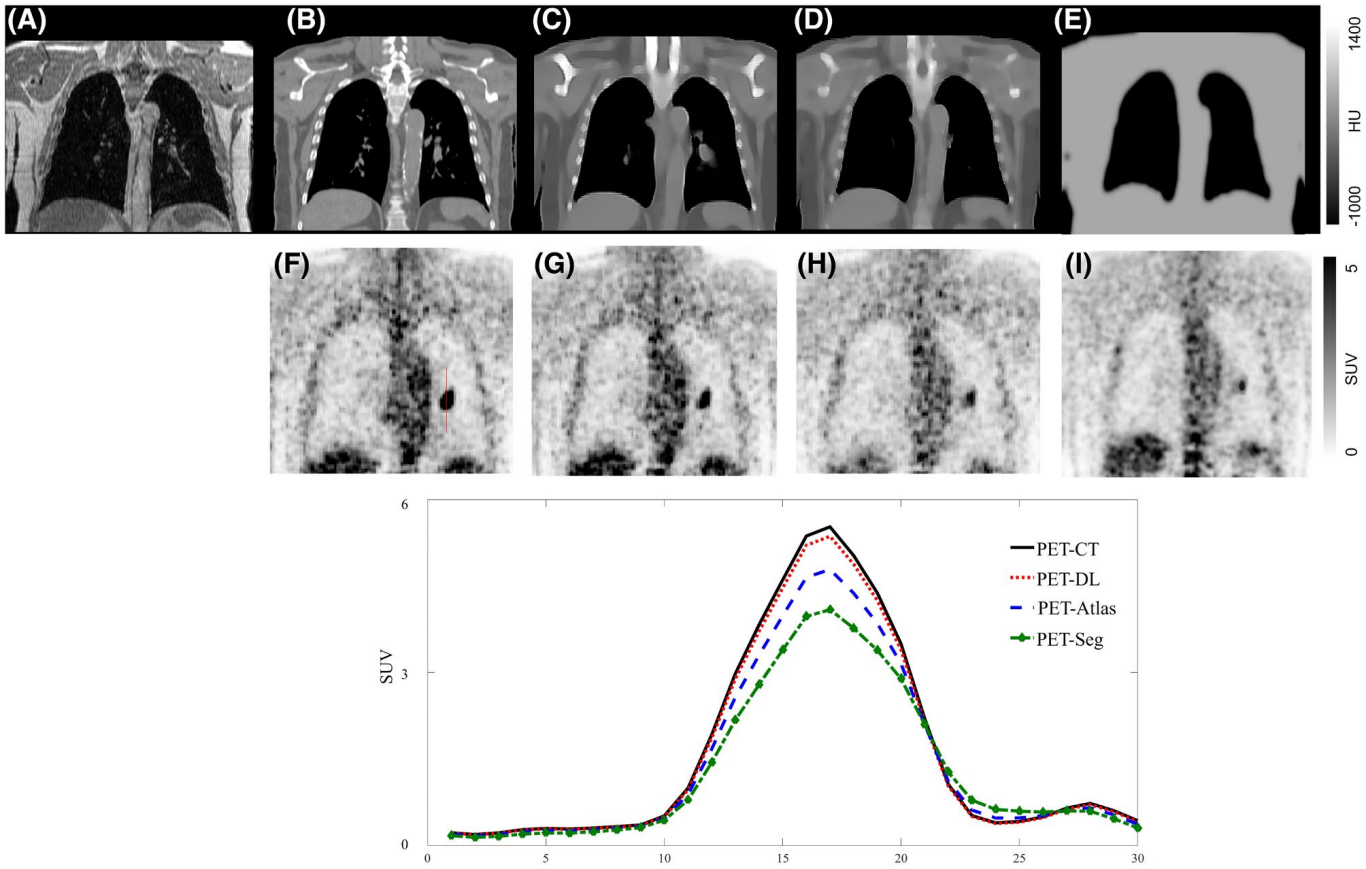
A clinical study presenting with small malignant lung nodule. Target MRI (A), reference CT image (B), sCT images generated using the deep learning (C), atlas‐based (D), and segmentation‐based (E) methods. PET‐CT (F), PET‐DL (G), PET‐Atlas (H), and PET‐Seg (I). The lower panel depicts vertical profiles drawn on the lung lesion in the different PET images

Figure 7 illustrates a patient with small malignant lung tumor visible on the PET/CT image. The lesion is reflected in the CT images as a patch of soft‐tissue in the lung, which is also discernible on MRI. Due to the small size of this structure, atlas‐, and segmentation‐based methods missed this lesion. Hence, the resulting sCTs images lack the corresponding patchy soft‐tissue structure in the lung leading to noticeable SUV bias in the lesion. Conversely, the deep learning approach was able to detect this structure from the MR image and correctly translate it into the resulting sCT image. As such, the PET‐DL image bears minimal bias in this region (plot over the lesion in Figure 7) compared to the reference PET/CT images. The mean SUV bias calculated within the VOI drawn on the lung lesion in Figure 7 was −3.1%, −18.7%, and −28.1% for the PET‐DL, PET‐Atlas, and PET‐Seg images, respectively. The other cases with small lung lesions and particularly lung edema are observed less frequently in torso PET/MRI. Hence, the cases reported in Figures 6 and 7 are the only ones of this kind observed in our torso PET/MR/CT dataset.

## 4 DISCUSSION

The overall assessment of quantification bias of radiotracer uptake when using PET images corrected for attenuation using the different sCT images (averaged over 25 subjects) demonstrated the superior performance of the deep learning approach. The residual convolutional network was able to detect and translate the most important structures from the MR images resulting in promising sCT images. This network was validated in a number of previous studies.^19^, ^20^, ^22^, ^30^ The deep learning method enabled the generation of patient‐specific sCT images wherein the full anatomical details in the MR images were translated into the sCT images. The performance of the deep learning model developed in this work is comparable with similar studies conducted on whole‐body/torso images. Klaser et al introduced imitating learning for MRI‐guided PET AC, wherein MRI to CT synthesis model also accounts for PET reconstruction errors.^34^ They reported an overall PET reconstruction error of 4.74% ± 1.52% compared to an atlas‐based method with an average error of 6.68% ± 2.06%. Armanious et al set out to generate synthetic CT images from PET nonAC images using a GAN model.^45^ Quantitative analysis revealed an SUV bias of −0.8% ± 8.6% across all organs (ranging from −30.7% to 27.1%) and 0.9% ± 9.2 (ranging from 19.6% to 29.2%) for malignant lesions.

Atlas‐based methods are capable of generating reasonable sCT images not only in brain imaging^16^, ^39^ but also in whole‐body/torso imaging wherein higher anatomical variability across the patients would challenge this approach.^9^, ^10^, ^46^, ^47^ However, since the atlas‐based method relies on the performance of image registration algorithms, this method could only translate the main anatomical structures from MR into sCT images but not the fine structures. This is partly due to the fact that these algorithms are not able to perfectly align all anatomical structures and the prior knowledge from the atlas dataset, which may not contain the same anatomical variations. Local anatomical variations are commonly overlooked in this approach, particularly when the input subject bears some sort of abnormalities.

The segmentation‐based method is inherently restricted by the bulk tissue classification wherein apart from tissue segmentation errors, the assignment of predefined linear attenuation coefficients to tissue classes does not allow this approach to offer patient‐specific solutions.^3^, ^48^ Apart from the sub‐optimal performance of segmentation‐ and atlas‐based methods in the definition of the anatomical details in the sCT images (compared to the deep learning method), they are not able to provide subject‐specific attenuation coefficients for the different tissues since the segmentation‐based method relies on predefined values whereas the atlas‐based method relies on the average of a population. In this regard, the deep learning method exhibited promising performance to generate not only sCT images with appropriate anatomical details but also with relatively accurate attenuation coefficients leading to minimal SUV bias in the corresponding PET images.

Overall, the deep learning algorithm exhibited promising results. However, this finding was already expected before conducting this study. Therefore, we focused on special cases, such as metal and body truncation artifacts, which are less considered when using deep learning solutions. When the input MR image suffers from body truncation or metal artifacts, a significant portion of the image is missing or skewed, which challenges the deep learning method with respect to extracting meaningful features and estimating the underlying anatomical structures. This issue would be more serious/severe when the training of the network is carried out with a dataset consisting of normal cases in majority (such as in this study). Conversely, atlas‐based methods are able to take into account the missing part of the input MR image through relying on the prior knowledge in the form of atlas dataset. In the case of metal artifacts, it is unlikely that the atlas‐based method could detect metal objects in sCT images. Nevertheless, since most of the images in the atlas dataset contain bony structures in the site of metal implants, a reasonably accurate sCT would be generated where the metal implant is replaced with bone tissue (Figure 4D). The advantage of the atlas‐based method over the deep learning approach was only in cases of severe metal artifact and body truncation, wherein a substantial region of the body was missing in the input MR images. The atlas‐based approach was capable of estimating/filling efficiently the missing regions and generating decent synthetic CT images owing to exploitation of prior knowledge in the form of atlas images. Apart from these cases, the atlas‐based method was substantially outperformed by the deep learning approach.

Although the deep learning approach exhibited sub‐optimal performance in cases of severe metal and body truncation artifacts, promising performance in terms of generating sCT images for abnormal anatomies (Figure 6) and small critical anatomical details (Figure 7). The training of the deep learning approach was performed with normal anatomy patients, and the patient in Figure 6 was completely unseen by the model. Nevertheless, since the input image contained distinguishable anatomical details and strong signals at the boundaries of organs (contrary to the metal and body truncation artifacts), the deep learning method was able to offer optimal (patient‐specific) solution relying on the available information in the input MR images. On the other hand, since there were no similar cases like the patients shown in Figures 6 and 7 in the atlas dataset, the atlas‐based method only offered a solution based on the average of the population in the atlas dataset, which is far from the optimal solution.

The deep learning approach exhibited overall superior performance, and in particular, for the anatomical abnormalities/nuances. However, in the case of severe metallic artifacts and body truncations, the atlas‐based method offered better solutions compared to those of the deep learning method. To account for these shortcomings/drawbacks of the deep learning method, three different strategies/solutions could be adopted/proposed. First, prior knowledge from an atlas‐based solution or shape models could be incorporated into deep learning to aid the estimation of the missing/corrupted regions. Second, prior to synthetic CT generation, a specialized network could be exploited to detect and correct metal artifacts and body truncations in MR images to avoid their detrimental effects on the resulting synthetic CT (such as the object completion framework proposed in Ref. [49]). Third, dedicated deep learning‐based synthetic CT generation architectures could be developed that model these corruptions within synthetic CT estimation process (either in input, feature layers, or reconstruction layers) to offer realistic solutions.

The deep learning approach offers promising solution for the generation of sCT from MR images in the context of PET/MRI AC or MRI‐only radiation treatment planning. However, the susceptibility of this approach to void area in MR images due to metal‐implants or body truncation should be particularly addressed. A possible solution to this challenge might be through training of the model with large similar samples containing metal and body truncation artifacts for the model to learn how to estimate the missing part of the input subject. Other solutions could be through application of a pre‐processing function on the input MR images, such as object completion algorithms^49^ to fill‐in the missing parts before feeding into the sCT generation model. This object completion step could also be applied on the resulting sCT images to correct the defects of the sCT generation model. PET images before AC (PET‐nonAC) also provide valuable information about the anatomical structures of patients, although in a lower resolution. Training the model with an additional channel for PET‐nonAC could address this issue as the correct body contour could be detected from PET‐nonAC images. However, this solution is only applicable in the context of PET/MR imaging and not in MRI‐only radiation treatment planning.

The three‐tissue class segmentation approach investigated in this work^28^ exhibited poor performance in the presence of body truncation and metal artifacts. However, the latest (advanced) versions of the segmentation‐based synthetic CT generation methods tend to accounted for void signals in MR images (due to metal artifacts or body truncation) through exploiting prior knowledge in the form body atlas and/or template.^26^ In this regard, Lindemann et al^50^ proposed B0 homogenization using gradient enhancement to provide an extension of the transaxial FOV in MR imaging. This approach would minimize the impact of body truncation on PET quantification. Using such a segmentation approach would reduce the sensitivity to the presence of metal and body truncation artifacts. Yet, the limitation of segmentation‐based approaches owing to their inability in providing patient‐specific attenuation distribution map still remains.

A major limitation of this study is the relatively small sample size (25 subjects). Although this number may be sufficient for the overall evaluation of the synthetic CT generation approaches, a comprehensive/conclusive assessment of the occurrence of outliers requires a far larger dataset. Moreover, the number of outliers was limited to few cases, which prevented drawing a generalized conclusion. Nevertheless, a key point in the training and/or performance assessment of deep learning approaches is the ratio/proportion of normal subjects to outliers. Increasing the number of training subjects may result in better performance of deep learning algorithms in terms of handling abnormalities/outliers provided the training dataset include a sufficient number of similar cases.

## 5 CONCLUSIONS

This study set out to compare the performance of the three commonly used MRI‐guided synthetic CT generation approaches, namely segmentation‐, atlas‐, and deep learning‐based approaches in the context of whole‐body/torso PET/MR imaging. Overall, the deep learning approach outperformed atlas‐ and segmentation‐based methods resulting in less than 4% tracer quantification bias across 25 patients. However, the primary focus of this work was on the study of the outliers. In case of sever truncation and metal‐artifacts in the input MR images, the deep learning approach was outperformed by the atlas‐based approach exhibiting suboptimal performance in the region affected by these artifacts. Conversely, for abnormal anatomies, such as the patient with only one lung or small malignant lesions in the lung, the deep learning method exhibited promising performance providing superior results compared to the other methods. It can be concluded that the deep learning‐based method provides promising solutions for synthetic CT generation from MRI. However, metal and body truncation artifacts should be specifically addressed.

## Supporting information


**FIGURE S1** Representative VOIs drawn on the abnormally increased radiotracer uptake in the head and neck area
**TABLE S1** Type and frequency of the special cases observed in the clinical studies
**TABLE S2** Quantitative accuracy of the estimated CT values for the different major tissue classes by the different deep learning models
**TABLE S3** Quantitative accuracy of the estimated CT values for the different major tissue classes by the different synthetic CT generation methods
