# Supplementary material for: MRI‐guided attenuation correction in torso PET/MRI: Assessment of segmentation‐, atlas‐, and deep learning‐based approaches in the presence of outliers
Source: Magn Reson Med. 2021 Sep 4;87(2):686–701. doi: 10.1002/mrm.29003 (PMC9292636; doi:10.1002/mrm.29003)
Supplement: Supplementary file 1 — FIGURE S1 Representative VOIs drawn on the abnormally increased radiotracer uptake in the head and neck area TABLE S1 Type and frequency of the special cases observed in the clinical studies TABLE S2 Quantitative accuracy of the estimated CT values for the different major tissue classes by the different deep learning models TABLE S3 Quantitative accuracy of the estimated CT values for the different major tissue classes by the different synthetic CT generation methods [file MRM-87-686-s001.pdf]

Supplemental Material

Supporting Information Table S1. Type and frequency of the special cases observed in the clinical studies.

**Metal artifact**	Dental filling<br>Shoulder prosthesis	4 patients<br>One patient
**Body truncation**	Missing arms	4 patients
**Abnormal anatomy**	Lung edema	One patient
**Anatomical nuance**	Small lung lesion	One patient

Supporting Information Table S2. Quantitative accuracy of the estimated CT values for the different major tissue classes by the different deep learning models.

	**Soft-tissue mean ± SD (Abs. mean ± SD)**	**Bone mean ± SD (Abs. mean ± SD)**	**Lung mean ± SD (Abs. mean ± SD)**	**Whole-body mean ± SD (Abs. mean ± SD)**
**ResNet (1)**	1±11 (21±7)	26±53 (57±31)	-23±59 (53±34)	11±29 (49±25)
**GAN (2)**	12±20 (33±12)	-45±70 (74±47)	43±65 (70±40)	32±47 (72±39)
**eCNN (3)**	-13±21 (37±15)	58±81 (87±54)	-57±72 (89±51)	-43±52 (83±47)
**Unet (4)**	-11±20 (31±12)	40±69 (70±46)	36±63 (61±37)	16±40 (62±33)
**Vnet (5)**	-10±19 (30±11)	39±67 (69±45)	34±62 (59±36)	15±39 (60±31)

Supporting Information Table S3. Quantitative accuracy of the estimated CT values for the different major tissue classes by the different synthetic CT generation methods.

	**Soft-tissue mean ± SD (Abs. mean ± SD)**	**Bone mean ± SD (Abs. mean ± SD)**	**Lung mean ± SD (Abs. mean ± SD)**	**Whole-body mean ± SD (Abs. mean ± SD)**
**DL-CT**	1±11 (21±7)	26±53 (57±31)	-23±59 (53±34)	11±29 (49±25)
**Atlas-CT**	-56±38 (72±33)	-91±70 (122±61)	48±66 (79±42)	-40±83 (109±67)
**Seg-CT**	-62±34 (73±31)	-620±91 (620±91)	71±73 (93±59)	-81±89 (176±70)

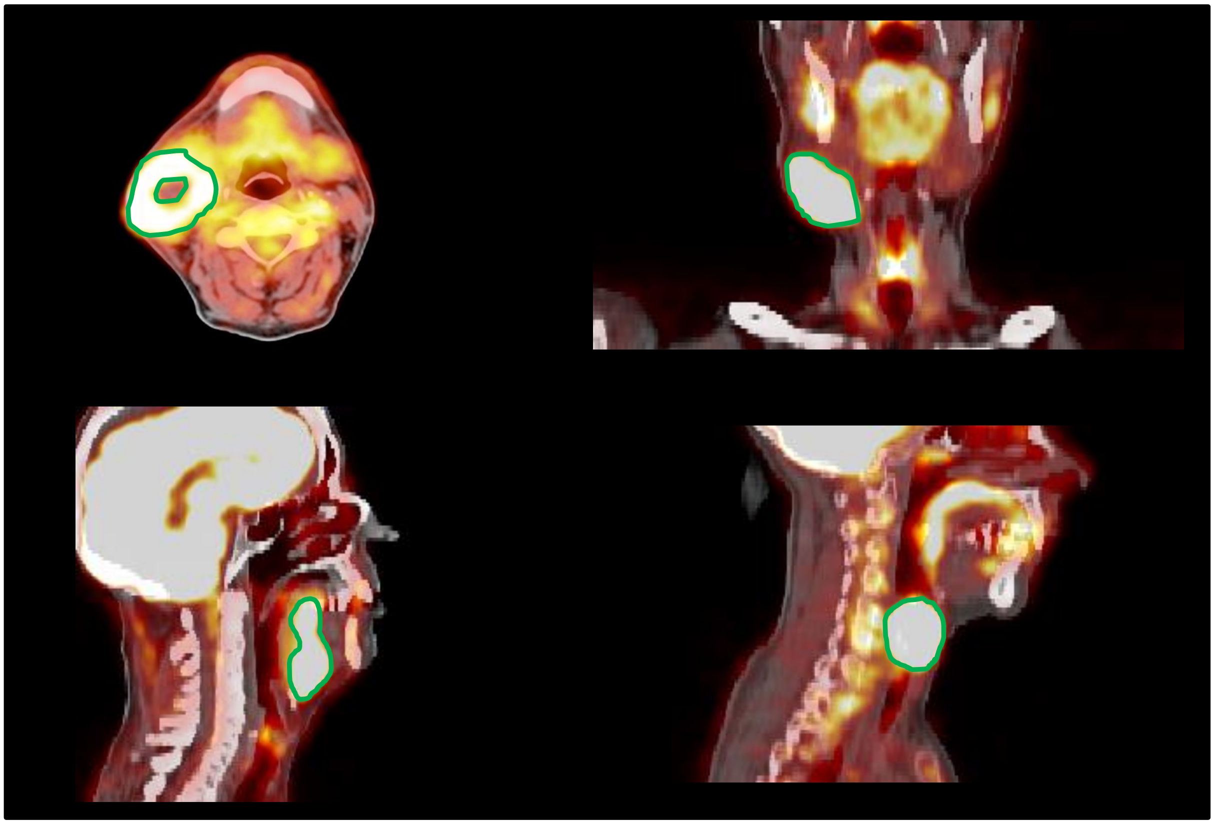

Supporting Information Figure S1. Representative VOIs drawn on the abnormally increased radiotracer uptake in the head and neck area.

References

1. Li W, Wang G, Fidon L, Ourselin S, Cardoso MJ, Vercauteren T. On the compactness, efficiency, and representation of 3D convolutional networks: brain parcellation as a pretext task. 2017. Springer. p 348-360.
2. Harms J, Lei Y, Wang T, Zhang R, Zhou J, Tang X, Curran WJ, Liu T, Yang X. Paired cycle-GAN-based image correction for quantitative cone-beam computed tomography. Med Phys 2019;46(9):3998-4009.
3. Bahrami A, Karimian A, Fatemizadeh E, Arabi H, Zaidi H. A new deep convolutional neural network design with efficient learning capability: Application to CT image synthesis from MRI. Med Phys 2020;47(10):5158-5171.
4. Çiçek Ö, Abdulkadir A, Lienkamp SS, Brox T, Ronneberger O. 3D U-Net: learning dense volumetric segmentation from sparse annotation. 2016. Springer. p 424-432.
5. Gibson E, Giganti F, Hu Y, Bonmati E, Bandula S, Gurusamy K, Davidson B, Pereira SP, Clarkson MJ, Barratt DC. Automatic Multi-Organ Segmentation on Abdominal CT With Dense V-Networks. IEEE Trans Med Imaging 2018;37(8):1822-1834.
